# A Traceable Low-Frequency Attenuation Standard from 1 kHz to 10 MHz for Next-Generation Wireless and EMC Calibration

**DOI:** 10.3390/s25196227

**Published:** 2025-10-08

**Authors:** Anton Widarta

**Affiliations:** Research Institute for Physical Measurement, National Metrology Institute of Japan, National Institute of Advanced Industrial Science and Technology, Tsukuba 305-8563, Japan; anton-widarta@aist.go.jp; Tel.: +81-50-3521-0882

**Keywords:** primary attenuation standard, traceability, inductive voltage divider (IVD), heterodyne detection, Type-B uncertainty (non-statistical), transfer standard attenuator, electromagnetic compatibility (EMC), wireless communication, low-frequency measurement

## Abstract

The growing demand for traceable, high-precision attenuation measurements in electromagnetic compatibility (EMC) testing and low-frequency wireless communication systems has driven the development of a primary attenuation standard covering 1 kHz to 10 MHz. The system employs a dual channel null-detection method using an inductive voltage divider (IVD) as a reference, ensuring the highest accuracy and traceability while eliminating sensitivity to detector nonlinearity. Attenuation at 1 kHz, 9 kHz, and 10 kHz is measured directly against the IVD ratio, while higher-frequency measurements (100 kHz–10 MHz) are performed via heterodyne detection, down-converting signals to 1 kHz for comparison. To ensure comparable accuracy at higher attenuation levels, a double-step method is applied at 9 kHz and 10 kHz to mitigate the increased IVD uncertainty above 1 kHz. Linearity is ensured by suppressing common-mode currents with toroidal ferrite chokes and minimizing inter-channel coupling. Type B (non-statistical) measurement uncertainties are evaluated, with major contributions from the IVD reference, system errors, and mismatch. The expanded uncertainties are 2.2 × 10^−3^ dB at 20 dB, 3.0 × 10^−3^ dB at 40 dB, and 4.0 × 10^−3^ dB at 60 dB attenuation. To facilitate wider dissemination and extend the calibration range, a resistive step attenuator with 10 dB pads is evaluated as a practical transfer standard, providing a simple and robust solution for traceable attenuation calibration in this frequency range.

## 1. Introduction

Attenuation is a fundamental parameter in characterizing the transmission of electromagnetic signals in radio frequency (RF) and microwave (MW) networks, playing a critical role in communication systems, sensor networks, and electromagnetic compatibility (EMC) testing environments [1,2,3,4,5,6,7,8,9,10,11]. National Metrology Institutes (NMIs) worldwide have established traceable attenuation standards, typically spanning from a few megahertz up to 40 GHz or 50 GHz [12,13,14,15,16,17,18,19,20], enabling accurate calibration of devices used in applications such as wireless communications, radar, and navigation systems.

With the rapid expansion of wireless technologies, particularly in vehicle radar, airport security imaging, and high-speed data communications, the demand for traceable measurements has extended into both the millimeter-wave and low-frequency regions. In response, leading NMIs, including the National Metrology Institute of Japan (NMIJ), have expanded their calibration capabilities beyond 50 GHz [21,22,23]. However, an equally critical gap remains at lower frequencies. Recent EMC regulations mandate traceability for measurements starting from 9 kHz, highlighting the urgent need for reliable and accurate attenuation standards in the 1 kHz to 10 MHz range [24,25]. Despite the significance of this frequency band for compliance testing and low-frequency wireless communication systems, there is, to the best of the authors’ knowledge, a notable lack of technical studies concerning the development and establishment of primary attenuation standards within this range [26,27]. Moreover, no key or bilateral comparison, typically employed to demonstrate the international equivalence of national measurement standards, has yet been conducted [28].

This paper presents a primary attenuation standard operating in the 1 kHz to 10 MHz frequency range, developed to meet emerging requirements in EMC and low-frequency wireless communication systems. The principle of the system was described in a previous conference paper [29]; here, the focus is on evaluating its uncertainty through measurement experiments and on proposing a step attenuator as an accurate working standard. The system employs a null-detection method realized via the IVD reference, which inherently provides high accuracy and robust traceability while minimizing measurement errors from detector nonlinearity. Measurements at 1 kHz, 9 kHz, and 10 kHz are directly referenced to the IVD voltage ratio, while signals from 100 kHz to 10 MHz are measured via heterodyne down-conversion to 1 kHz. To ensure comparable accuracy at higher attenuation levels, a double-step method [30] is applied at 9 kHz and 10 kHz to mitigate the increased IVD uncertainty above 1 kHz. Stable linearity is achieved by suppressing common-mode currents with toroidal ferrite chokes and minimizing inter-channel coupling. Type B (non-statistical) uncertainty is rigorously evaluated, considering the IVD reference, system errors, and mismatch. The system achieves highly accurate attenuation measurements up to 60 dB, with excellent stability and sensitivity. By evaluating a commercial RF resistive step attenuator with 10 dB pads as a practical transfer standard, the technique enables robust, traceable calibration beyond 100 dB, providing a simple and reliable solution for low-frequency RF measurements suitable for broader metrology and industrial applications.

## 2. Measurement System

### 2.1. Primary Standard

**Figure 1:**
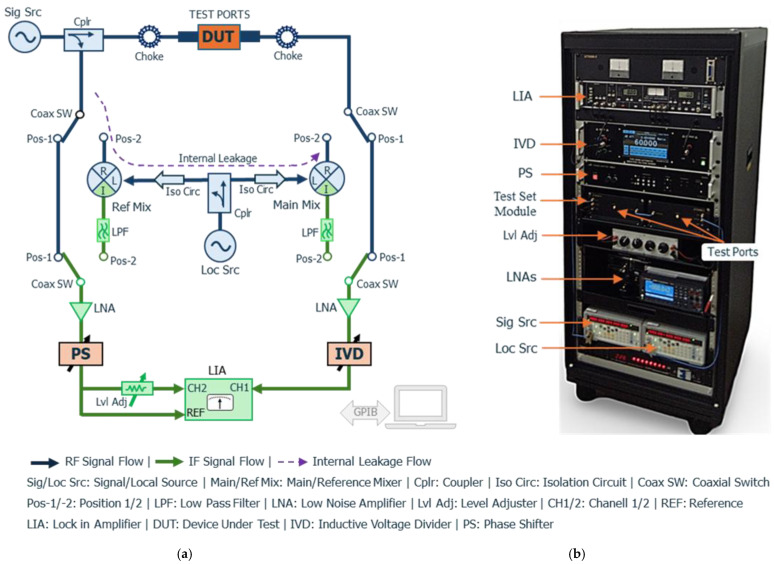
Proposed attenuation measurement system: (**a**) block diagram and (**b**) photograph. The system uses a dual-channel null-balancing technique with an IVD as the reference, covering 1 kHz to 10 MHz. Subrange 1 (1 kHz, 9 kHz, 10 kHz): direct comparison with the IVD. Subrange 2 (100 kHz–10 MHz): comparison with the IVD after down-conversion to 1 kHz. In the block diagram, the RF section is shown in blue, the IF section in green, and key components—DUT, IVD, and phase shifter—are highlighted in red.

Figure 1 presents a simplified block diagram (a) and a photograph (b) of the proposed precision attenuation measurement system. The system operates over 1 kHz to 10 MHz and employs a dual-channel null-detection technique with a seven-decade IVD as the reference standard. It is designed to achieve high accuracy, with a measurement capability of up to 60 dB attenuation. The frequency range is divided into two subranges: Subrange 1 (1 kHz, 9 kHz, and 10 kHz), where attenuation is directly compared with the IVD, and Subrange 2 (100 kHz to 10 MHz), where measurements are performed after down-conversion to 1 kHz using heterodyne detection. This configuration ensures consistent signal processing and high precision across both subranges.

#### 2.1.1. Measurement Principle in Subrange 1 (1 kHz, 9 kHz, 10 kHz)

For Subrange 1, the RF signal from the source (Model SG382) is split into measurement and reference paths via a directional coupler (Model ZFDC-10-6-S). The measurement signal passes through the Device Under Test (DUT), mounted between two test ports, each equipped with a 10 dB matching pad to minimize mismatch uncertainties and a toroidal ferrite choke to suppress common-mode currents, which are particularly significant at low frequencies. The output signal from the DUT enters a low-noise preamplifier (LNA, Model T-01LNA), which also acts as a buffer to isolate the IVD (Model 6415A). After amplification, the signal passes through the IVD and is routed to Channel 1 (CH1) of a lock-in amplifier (LIA, Model 5210), serving as a high-resolution null detector. Simultaneously, the reference signal is amplified, phase-adjusted via a phase shifter (PS, Model 5920A), and split into two branches. One branch is applied to the LIA reference (REF) input for synchronization, while the other passes through a level adjuster before reaching Channel 2 (CH2). The IVD voltage ratio and PS phase shift are adjusted before and after inserting the DUT to achieve a null balance between CH1 and CH2. Letting the IVD settings before and after DUT insertion be *S_i_* and *S_f_*, respectively, the attenuation *A* in decibels is given by

(1) $$A=-20\;{log}_{10}\left(\;\frac{S_{i}}{S_{f}}\right).$$

#### 2.1.2. Measurement Principle in Subrange 2 (100 kHz–10 MHz)

For Subrange 2, attenuation measurements are performed using an intermediate frequency (IF) substitution technique. In this method, both the measurement and reference signals are down-converted to 1 kHz by the main and reference mixers (Model M1XCA), driven by a common local oscillator (Model SG382). The oscillator signal is split via a second directional coupler of the same model, and the system switches (Figure 1) are set to position 2 to activate this measurement mode. To ensure high channel isolation and suppress crosstalk (internal leakage), two amplifier (Model ZFL-500)–attenuator assemblies are placed between the reference mixer and the second directional coupler, and between the main mixer and the second directional coupler. Each assembly comprises a 20 dB gain amplifier providing 40 dB of isolation, followed by a level-adjuster attenuator, yielding at least 60 dB of isolation per path. Considering the mixer R-L port isolation (>30 dB), the 40 dB directivity of the directional coupler, the total system isolation is estimated to exceed 160 dB. The resulting IF signals are filtered through low-pass filters (LPFs) to remove high-frequency components and ensure signal purity and then processed using the same null-detection scheme as Subrange 1, with the IVD and LIA.

This modular configuration enables accurate and traceable attenuation measurements across the underrepresented low-frequency RF range. By applying consistent signal processing principles in both direct and heterodyne detection modes, the system ensures high measurement integrity over a broad frequency span.

### 2.2. Transfer Standard

**Figure 2:**
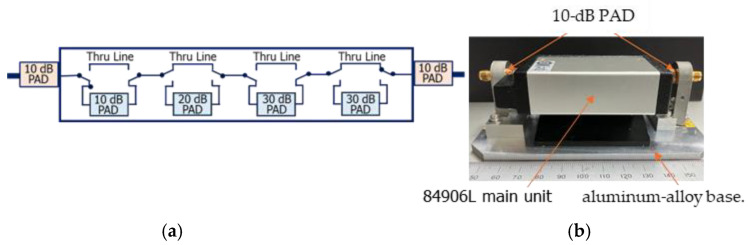
A commercial programmable RF resistive step attenuator (Model 84906L) proposed as the transfer standard: (**a**) schematic diagram illustrating the internal configuration with 10 dB pads attached at each port; (**b**) photograph of the exterior, showing the main unit of the step attenuator, the attached 10 dB pads, and the aluminum-alloy base.

As shown in Figure 2, the proposed transfer standard is a commercial programmable RF resistive step attenuator (Model 84906L). The figure includes both a schematic diagram of the internal configuration with 10 dB pads attached at each port and a photograph of the exterior, showing the attenuator unit, the attached pads, and the aluminum-alloy base. The attenuator was modified by adding 10 dB pads at both ports to minimize mismatch errors and was mounted on an aluminum-alloy base to improve mechanical stability and robustness. The transfer standard was then calibrated and evaluated using the primary attenuation measurement system described above. Precision RF resistive attenuators typically exhibit a flat frequency response below several tens of megahertz, then a single-frequency calibration (e.g., at 1 MHz) is sufficient to characterize the entire range from 1 kHz to 10 MHz. In combination with a suitable measurement receiver [31] and the double-step attenuation technique [30], this approach provides a simple, robust, and traceable method for extending calibration capabilities to attenuation levels exceeding 100 dB. Importantly, the use of such a transfer standard offers a practical means to disseminate the primary standard to routine calibration services and support a wider range of measurement applications.

## 3. Measurement Experiment and Uncertainty Evaluation

### 3.1. Primary Standard Evaluation

This section presents a comprehensive uncertainty analysis of the proposed attenuation measurement system. Type B (non-statistical) uncertainty is categorized into three main components: IVD reference standard; system-related errors, including resolution, noise, drift, and nonlinearity; as well as the contribution from the gauge block attenuator (GBA), which will be discussed in more detail below in the context of the double-step measurement technique and mismatch. All uncertainties were evaluated in accordance with the Guide to the Expression of Uncertainty in Measurement (GUM) [32,33].

(1) *IVD Reference Standard*

The IVD is a precision autotransformer that produces highly accurate voltage ratios using mutual inductance and the winding turns ratio. In this system, a metrology-grade seven-decade IVD, made of seven cascaded stages of ten-part transformers, is used. This design provides extremely stable voltage division at the part-per-million (ppm) level, allowing very small attenuation resolutions, down to 0.00001 dB at 20 dB and 0.001 dB at 60 dB. The manufacturer specifies its accuracy as 0.5 ppm at 1 kHz (0.00005 dB at 20 dB) and 50 ppm at 10 kHz (0.005 dB at 20 dB). To ensure traceability and reliable uncertainty estimation, the IVD was calibrated against the Japan national standard for AC voltage ratios at 1 kHz and 10 kHz, which serves as the system’s reference. This combination of precision and traceability makes the IVD an ideal reference standard for attenuation measurements. If the maximum calibration uncertainty of the IVD is denoted by *σ*, the measured attenuation *A*′ in decibels can be expressed as

(2) $$A^{’}=-20\;{log}_{10}\left(\;\frac{S_{i}\;\pm \;\sigma }{S_{f}}\right).$$

From Equations (1) and (2), the bound uncertainty, Δ*A*, of the measured attenuation in decibels due to *σ* is expressed as:

(3) $$∆A=A^{’}-A=-20\;{log}_{10}\left(\;\frac{1\pm \sigma }{S_{i}\;}\right)\approx \mp \;8.686\frac{\sigma }{S_{i}}\;\;,\;(\sigma \ll S_{i}).$$

According to the calibration certificate, *σ* is 0.1 ppm (1.0 × 10^−7^) at 1 kHz, increasing to 3 ppm (3.0 × 10^−6^) at 10 kHz. Using Equation (3), the corresponding attenuation limits *Δ*A** are calculated and summarized in Table 1. *Δ*A** at 10 kHz is approximately 100 times larger than at 1 kHz.

**Figure 3:**
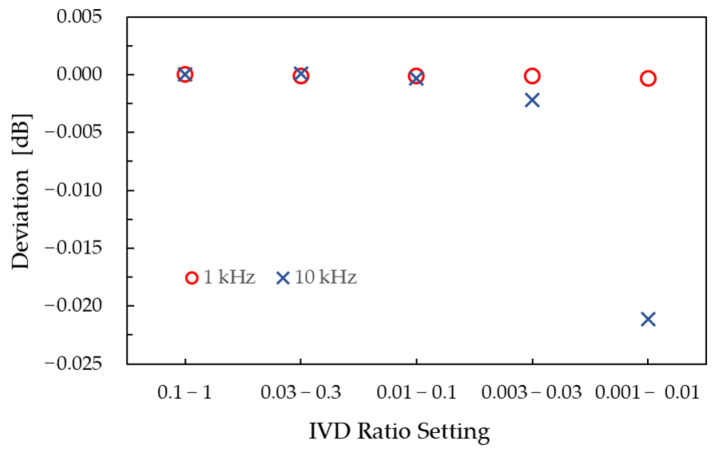
Validation of the IVD calibration uncertainty estimation through 20 dB attenuation measurements using different IVD settings.

To validate this uncertainty estimation, measurements of a nominal 20 dB step attenuation were performed using five different IVD ratio settings: *S_i_* = 0.1 to *S_f_* = 1, 0.03 to 0.3, 0.01 to 0.1, 0.003 to 0.03, and 0.001 to 0.01. By applying these different ratio settings to realize the same attenuation value, the effect of IVD calibration uncertainty can be directly assessed against the theoretical estimates. The results are presented in Figure 3. At 1 kHz (blank circles), the measurements remain stable across all IVD settings, indicating a negligible contribution from the IVD ratio. In contrast, at 10 kHz (crosses), a measurable deviation is observed, particularly for the *S_i_* = 0.001 to *S_f_* = 0.01 setting, where the measured difference reaches approximately 2.2 × 10^−2^ dB. This deviation is consistent with the estimated uncertainty of the IVD at these settings at 10 kHz (Table 1).

To reduce this deviation for attenuations above 40 dB at 10 kHz (and similarly at 9 kHz), a double-step technique [30] is employed: first, the IVD is set from 0.1 to 1 (step 1: 0–20 dB) as a GBA with Δ*A* = 2.6 × 10^−4^ dB; then from 0.01 to 1 (step 2: 20–60 dB) with *Δ*A** = 2.6 × 10^−3^ dB. This approach provides an approximate tenfold improvement over the single-step method. However, the uncertainty introduced by the GBA, which is discussed below, must also be considered.

Finally, to derive the standard uncertainty due to this IVD calibration, *u*(*X*_1_), half of each *Δ*A** value was taken and divided by $\sqrt{3}$, assuming a uniform probability distribution. The resulting values are listed in Table 2.

(2) *System Resolution and Noise*

**Figure 4:**
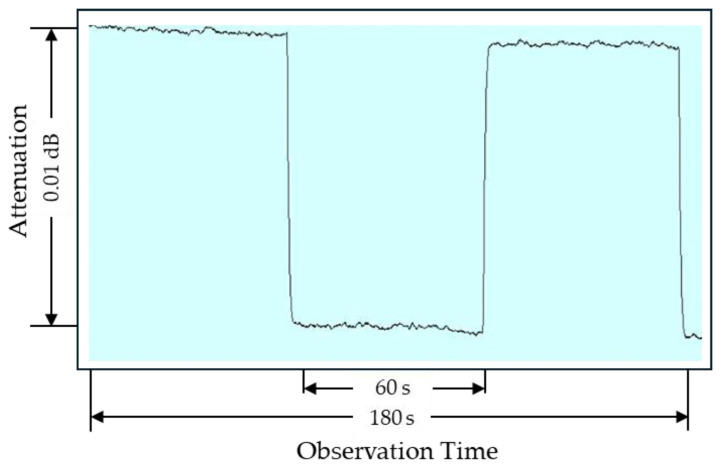
Balance state of the measured signal with a 20 dB DUT at 9 kHz, showing system stability for noise-related uncertainty estimation.

The system resolution and noise was evaluated by monitoring the balance state of the signals at the LIA. Figure 4 shows the results at 9 kHz with a DUT attenuation of 20 dB. After 60 s of observation, the IVD setting was adjusted to introduce a 0.01 dB change, allowing the signal behavior to be clearly distinguished. The fluctuations observed in this figure indicate the influence of system noise, with amplitudes of approximately ±5 × 10^−4^ dB. The standard uncertainty due to system resolution and noise, *u*(*X*_2_), was determined by taking half of the observed fluctuation range and assuming a uniform probability distribution, giving a divisor of $\sqrt{3}$. The resulting value is estimated to be 1.4 × 10^−4^ dB. For DUT attenuations of 40 dB and 60 dB, *u*(*X*_2_) was similarly estimated to be 2.9 × 10^−4^ dB and 8.7 × 10^−4^ dB, corresponding to fluctuation ranges of 1 × 10^−3^ dB and 3 × 10^−3^ dB, respectively.

(3) *Drift*

The system drift was also evaluated by monitoring the balance state of the signals at the LIA over 60 s, corresponding to the maximum measurement duration. As shown in Figure 4, the drift was less than 0.001 dB. The associated standard uncertainty due to system drift, *u*(*X*_3_), was therefore estimated as 2.9 × 10^−4^ dB, assuming a uniform probability distribution.

(4) *Nonlinearity*

**Figure 5:**
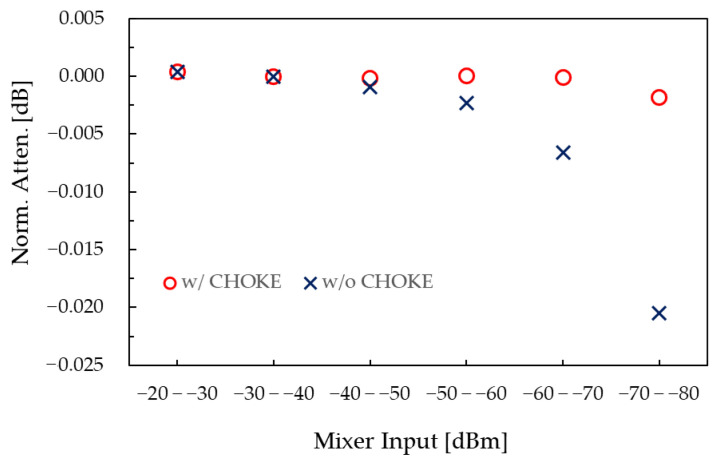
Linearity of the attenuation measurement system at 1 MHz.

The measurable attenuation range, or dynamic range, of the system is mainly limited by its linearity. Figure 5 shows the linearity results at 1 MHz. Blanck circles indicate measurements obtained using a variable attenuator in 10 dB steps, with the preamplifier input swept from −20 dBm to −80 dBm. Measured attenuation values are normalized to that at −30 dBm. The observed nonlinearities are 6.0 × 10^−4^ dB for a measurement range up to 20 dB (−20 to −40 dBm), 1.0 × 10^−3^ dB for up to 40 dB (−20 to −60 dBm), and 2.9 × 10^−3^ dB for 60 dB (−20 to −80 dBm). These results include uncertainties due to mixer and preamplifier nonlinearities, as well as leakage and common-mode effects.

Leakage can be classified as internal or external. Internal leakage propagates only through the coaxial line as indicated by the dashed line in Figure 1, whereas external leakage travels via paths outside the coaxial line. The effects of both leakage types depend on the relative phase between the measurement signal and the leakage signal at the main mixer input. If we let the signals arriving at the main mixer through the DUT and the leakage paths have amplitude of *S* and *L*, respectively, then the maximum uncertainty *ΔA_L_* due to the leakage in the attenuation measurements can be expressed in dB by:

(4) $$∆A_{L}=-20\;\log_{10}{\left(1\pm \frac{L}{S}\right)}^{-1}$$

As described in Section 2.1.2., the system isolation is expected to exceed 160 dB, comprising 30 dB from the reference mixer isolation, 60 dB from the isolation circuit assembly, 40 dB from the directivity of the second coupler, and 30 dB from the main mixer isolation. Using Equation (4), the resulting uncertainty due to internal leakage is 8.7 × 10^−5^ dB at 60 dB, corresponding to a leakage-to-signal ratio (*L*/*S*) of 1 × 10^−5^. This contribution is negligible compared with the linearity results.

Unlike internal leakage, the attenuation of external leakage paths is difficult to estimate because it depends on uncontrolled propagation conditions. In this study, external leakage effects were evaluated by comparing measurement values at a 60 dB level under relative phase conditions of 90°, 180°, and 270°. The observed differences between the measured values can be neglected, as they remain below the nonlinearity value at 60 dB estimated above.

To demonstrate the effectiveness of toroidal ferrite chokes on the system’s test ports in suppressing common-mode currents, linearity measurements were also performed with the toroidal ferrite chokes removed. The results, shown as crosses in Figure 5, reveal noticeable differences starting at a mixer input level of −50 dBm, increasing as the input decreases. At −80 dBm, the difference exceeds 0.02 dB.

Finally, the standard uncertainties due to nonlinearity, *u*(*X*_4_), were estimated by taking half of the observed linearity range and applying a uniform distribution with a divisor of $\sqrt{3}$. The resulting values are 1.7 × 10^−4^ dB for 20 dB, 2.9 × 10^−4^ dB for 40 dB, and 8.4 × 10^−4^ dB for 60 dB.

(5) *Gauge Block Attenuato* *r (GBA)*

In the double-step technique, for example, a 60 dB setting of a variable attenuator is measured in two steps. The first step involves measuring the attenuation from the 0 dB to 20 dB position, while the second step covers the range from 20 dB to 60 dB. The standard uncertainty associated with the gauge block, *u*(*X*_5_), corresponds to the combined standard uncertainty of the first-step measurement result, excluding the contribution from mismatch.

(6) *Mismatch*

If an attenuation measurement is carried out without the source and load being perfectly matched, there will be an error, i.e., a mismatch error, in the result. In high-precision attenuation measurements, this mismatch is often the largest term contributing to the systematic uncertainty. For a variable attenuator as a DUT, the mismatch uncertainty *σ_M_* is calculated by substituting S-parameter (*S_ij_*) of the DUT and reflection coefficients (*Γ_G_*, *Γ_L_*) of the test ports measured into the next expression [34].

(5) $$\sigma_{M}=\frac{8.686}{\sqrt{2}}{\left\{\;{\left|\Gamma_{G}\right|}^{2}{\left|S_{11i}-S_{11f}\right|}^{2}+{\left|\Gamma_{L}\right|}^{2}\left|S_{22i}-S_{22f}\right|\right.}^{2}\;\;\;\;\;\;\;\;\;\;\;\;\;\;{\left.+{\left|\Gamma_{G}\right|}^{2}{\left|\Gamma_{L}\right|}^{2}{\left|S_{21i}^{2}-S_{21f}^{2}\right|}^{2}\right\}}^{1/2}$$

where *i* and *f* denote the initial and final states of the DUT, respectively.

By placing precision 10 dB pads at the test ports, *Γ_G_* and *Γ_L_* were reduced to below 0.01. The pads effectively lowered both the equivalent source reflection and the reflections from the main mixer or LNA from approximately 0.1 to 0.01. For the DUT, a resistive step attenuator exhibits good impedance matching (|*S_ii_*| ≦ 0.01) in this low-RF range, resulting in a standard uncertainty due to mismatch, *u*(*X*_6_), of 1 × 10^−3^ dB (U-shaped distribution).

Table 3 summarizes the estimated uncertainties associated with attenuation measurements of a step attenuator (Model 84906L) at nominal attenuation values of 20 dB, 40 dB, and 60 dB. The standard deviation of the mean (SDOM) was determined from 10 repeated measurements. The expanded uncertainty is reported as the combined standard uncertainty multiplied by a coverage factor *k* = 2, corresponding to a coverage probability of approximately 95% for a normal distribution. The application of the double-step technique at 9 kHz and 10 kHz substantially reduces the expanded uncertainty for 60 dB measurements from 0.016 dB to 0.004 dB, reaching levels comparable to those achieved at 1 kHz and across the 100 kHz–10 MHz range. These expanded standard uncertainties are much lower than those of a recently developed overseas NMI system [27], which reports 5.0 × 10^−3^ dB at 10 dB, 5.0 × 10^−3^ dB at 30 dB, and 8.0 × 10^−3^ dB at 60 dB.

### 3.2. Transfer Standard Evaluation

**Figure 6:**
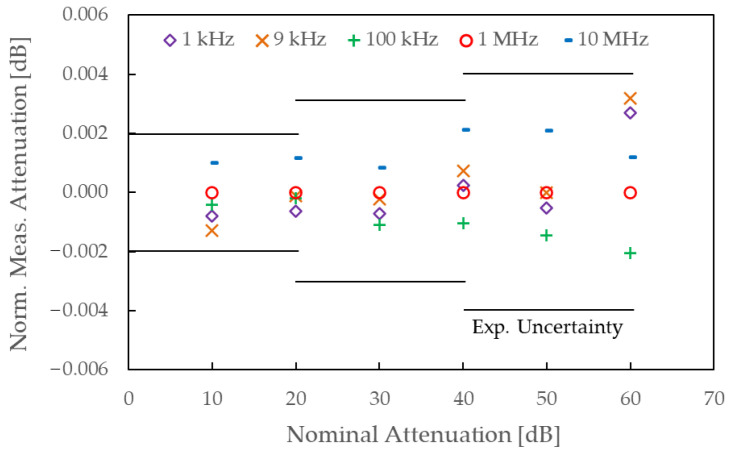
Measurement results of the step attenuator (model 84906L) with 10 dB pads mounted on both ports, evaluated as a candidate for the transfer standard.

Figure 6 shows the measurement results of the proposed transfer standard at nominal attenuation levels from 10 dB to 60 dB in 10 dB steps. Measurements were conducted at 1 kHz, 9 kHz, 100 kHz, 1 MHz, and 10 MHz. The data were normalized to the 1 MHz results and plotted to assess frequency dependence. As shown, deviations across frequency are minimal, around ±1.0 × 10^−3^ dB, ±2.0 × 10^−3^ dB, and ±3.0 × 10^−3^ dB for the 20 dB, 40 dB, and 60 dB settings, respectively, and remain within the expanded uncertainty limits indicated by the horizontal error bars. These results demonstrate that a single-frequency calibration at 1 MHz is sufficient to characterize the behavior of the step attenuator across the entire 1 kHz to 10 MHz range. Therefore, a working-standard system for attenuation calibration across this frequency range can be effectively implemented by directly comparing the device under test with a reference standard calibrated at a single frequency, i.e., 1 MHz, thereby simplifying the system configuration and eliminating the need for frequency conversion circuitry.

The combined standard uncertainties of this transfer standard are estimated following the approach described in Section 3 and are 4.8 × 10^−4^ dB, 9.7 × 10^−4^ dB, and 1.8 × 10^−3^ dB for the 20 dB, 40 dB, and 60 dB attenuation levels, respectively. These values include the standard uncertainty due to frequency response flatness, calculated as half of the maximum deviation observed in Figure 5 and divided by $\sqrt{3}$, assuming a uniform probability distribution. The contribution from mismatch is excluded, as 10 dB matching pads were attached to both ports of the attenuator, rendering its effect negligibly small. These uncertainties represent the contribution of the reference standard when establishing a working-standard system using a general-purpose receiver and, similar to the IVD in the primary system, constitute a minor component of the overall uncertainty.

Furthermore, with the double-step technique [30], this transfer standard enables attenuation calibrations above 100 dB, providing a practical and scalable solution for field calibration and traceability in low-frequency wireless and EMC applications.

### 3.3. System Validation

**Figure 7:**
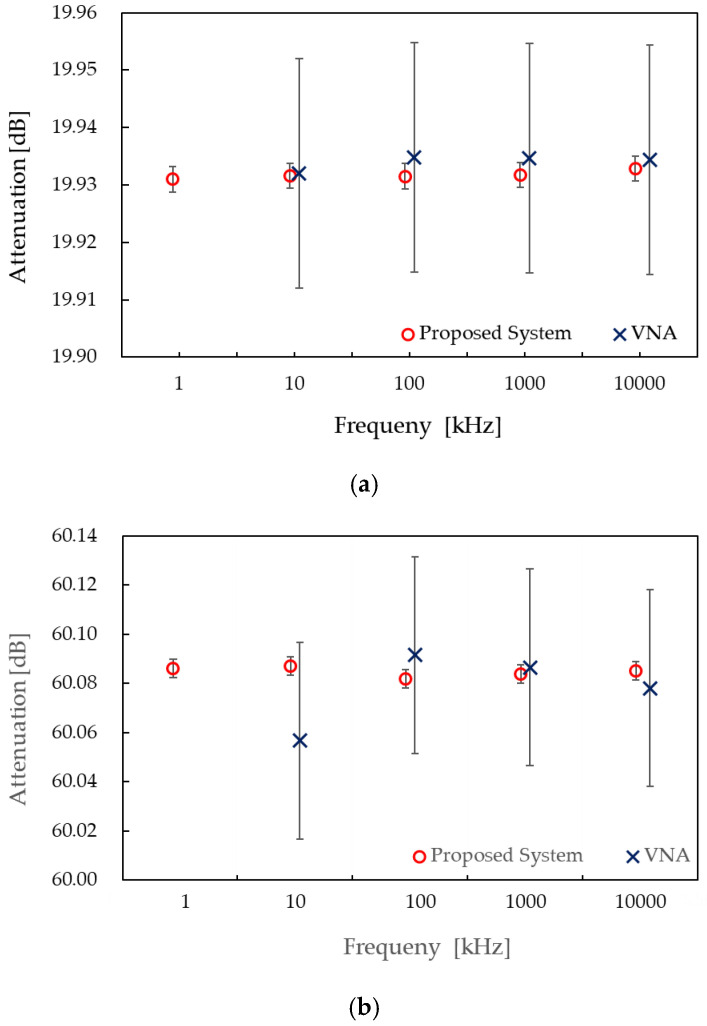
Measurement results of a resistive step attenuator (84906L) with nominal values of (**a**) 20 dB and (**b**) 60 dB in the frequency range of 10 kHz–10 MHz, obtained using the proposed primary system (circles) and a commercial VNA (crosses, error bars = U/5).

To verify the effectiveness of the proposed measurement system, RF resistive step attenuators (Model 84906L) with nominal values of 20 dB and 60 dB were measured at 1 kHz, 10 kHz, 100 kHz, 1 MHz, and 10 MHz. The results are shown in Figure 7(a),(b), where the circles represent the mean of ten repeated measurements and the vertical error bars indicate the expanded uncertainty as described above. Minimal variation was observed across the frequency range, consistent with the flat frequency response of the RF resistive attenuator within this low-RF range.

For comparison, measurements were also performed using a vector network analyzer (VNA: E5071C), shown by the crosses in Figure 7; the 1 kHz result is not included because the VNA measurement range begins at 9 kHz. The VNA measurement parameters were set as follows: input power of 0 dBm, IF bandwidth of 100 Hz, and averaging over 50 sweeps. Vertical error bars indicate the uncertainties evaluated according to the manufacturer’s software [35]; for visualization, they are displayed as U/5. Slight differences were observed between the two systems, but all remained within the VNA’s uncertainty budget. A more pronounced deviation was found for the 60 dB measurement at 10 kHz, the lowest frequency tested. This discrepancy is likely attributable to the VNA’s suboptimal handling of common-mode signals, which becomes increasingly significant at lower frequencies.

To achieve international recognition as a national standard, the system will undergo validation through bilateral comparisons with standards developed by other NMIs [26,27] and through participation in key comparisons organized under the BIPM framework.

## 4. Conclusions

A primary attenuation measurement standard covering 1 kHz to 10 MHz has been successfully developed to meet the growing traceability demands driven by EMC regulations and low-frequency wireless communication systems. By employing a null-detection method with an IVD reference, the system achieves the highest level of accuracy and traceability and remains unaffected by detector nonlinearity, ensuring its robustness as a national standard. Attenuation measurements at 1 kHz, 9 kHz, and 10 kHz are performed directly via IVD ratio comparisons, while measurements from 100 kHz to 10 MHz are down-converted to 1 kHz using heterodyne detection. To mitigate the increased IVD uncertainty above 1 kHz, a double-step measurement technique is applied, effectively reducing uncertainty for attenuations above 40 dB at 9 kHz and 10 kHz. Stable linearity better than 0.001 dB over a dynamic range exceeding 60 dB is achieved by suppressing internal and external leakage through robust channel isolation and effective component shielding. Toroidal ferrite chokes provide strong suppression of common-mode currents, particularly in the kHz range, with effects exceeding 0.02 dB. The resulting expanded uncertainties (*k* = 2) are 0.002 dB, 0.003 dB, and 0.004 dB for 20 dB, 40 dB, and 60 dB attenuation levels, respectively, and are substantially lower than those of recently developed overseas NMI systems.

A commercial step attenuator equipped with 10 dB pads has been validated as an effective transfer standard. Calibration at a single frequency (1 MHz) ensures accurate performance across the full 1 kHz–10 MHz range, enabling a simplified working-standard system without frequency conversion. With the double-step technique, the measurement range can be extended beyond 100 dB, supporting traceable and efficient calibration for a wide range of EMC testing, sensor, and communication applications.

To achieve international recognition as a national standard, the system will be validated through bilateral comparisons with standards developed by other NMIs and by participation in key comparisons organized under the BIPM framework.

## Figures and Tables

**Table 1:** Limits of attenuation measurement uncertainty, Δ*A*, due to the IVD calibration uncertainty, *σ*, at 1 kHz and 10 kHz.

Nominal Attenuation [dB]	IVD Setting		Frequency [kHz]	
			1	10
			Maximum Calibration Uncertainty of IVD *σ*	
			1.0 × 10^−7^	3.0 × 10^−6^
	*S_i_*	*S_f_*	Limit of Atten. Meas. Uncertainty *ΔA* [dB]	
20	0.1	1	8.7 × 10^−6^	2.6 × 10^−4^
40	0.01	1	8.7 × 10^−5^	2.6 × 10^−3^
60	0.001	1	8.7 × 10^−4^	2.6 × 10^−2^

**Table 2:** Standard uncertainty due to IVD calibration, *u*(*X*_1_), derived from the *Δ*A** by assuming a uniform distribution with a divisor of $\sqrt{3}$

Nominal Attenuation [dB]	IVD Setting		Frequency [Hz]	
			1	10
	*S_i_*	*S_f_*	Limit of Atten. Meas. Uncertainty *ΔA* [dB]	
20	0.1	1	2.5 × 10^−6^	7.5 × 10^−5^
40	0.01	1	2.5 × 10^−5^	7.5 × 10^−4^
60	0.001	1	2.5 × 10^−4^	7.5 × 10^−4^

**Table 3:** Summary of the uncertainty estimation for attenuation measurements.

Uncertainty Source		Cat.	Prob. Dis.	Frequency [Hz]					
				1 k, 100 k to 10 M			9 k, 10 k		
				Nominal Attenuation [dB]					
				20	40	60	20	40	60
				*u(X_i_)* [dB]	*u(X_i_)* [dB]	*u(X_i_)* [dB]	*u(X_i_)* [dB]	*u(X_i_)* [dB]	*u(X_i_)* [dB]
1	IVD Ref. Std.	B	Unif.	2.5 × 10^−6^	2.5 × 10^−5^	6.7 × 10^−4^	7.5 × 10^−5^	7.5 × 10^−4^	7.5 × 10^−4^
2	Sys. Res./Noise	B	Unif.	1.4 × 10^−4^	2.9 × 10^−4^	8.7 × 10^−4^	1.4 × 10^−4^	2.9 × 10^−4^	8.7 × 10^−4^
3	Drift	B	Unif.	2.9 × 10^−4^	2.9 × 10^−4^	2.9 × 10^−4^	2.9 × 10^−4^	2.9 × 10^−4^	2.9 × 10^−4^
4	Nonlinearity	B	Unif	1.7 × 10^−4^	2.9 × 10^−4^	8.4 × 10^−4^	1.7 × 10^−4^	2.9 × 10^−4^	8.4 × 10^−4^
5	Gauge Block	B	Unif.						3.7 × 10^−4^
6	Mismatch	B	U	1.0 × 10^−3^	1.0 × 10^−3^	1.0 × 10^−3^	1.0 × 10^−3^	1.0 × 10^−3^	1.0 × 10^−3^
7	SDOM	A	Norm.	1.0 × 10^−4^	6.0 × 10^−4^	8.0 × 10^−4^	1.0 × 10^−4^	6.0 × 10^−4^	8.0 × 10^−4^
*u_c_*	Combine Standard Unc.			1.1 × 10^−3^	1.3 × 10^−3^	1.9 × 10^−3^	1.1 × 10^−3^	1.5 × 10^−3^	2.0 × 10^−3^
*U*	Expanded Uncertainty			2.2 × 10^−3^	2.6 × 10^−3^	3.8 × 10^−3^	2.2 × 10^−3^	3.0 × 10^−3^	4.0 × 10^−3^

Ref. Std.: Reference Standard. Sys. Res.: System Resolution. Unc.: Uncertainty. Cat.: Category. Prob. Dis.: Probability Distribution.

## Data Availability

The data presented in this study are available within the article.

## Abbreviations

The following abbreviations are used in this manuscript:

CH	Chanel
Coax SW	Co-axial Switch
Cplr	Coupler
DUT	Device Under Test
EMC	Electromagnetic Compatibility
GBA	Gauge Block Attenuator
GUM	Guide to the Expression of Uncertainty in Measurement
Iso Circ	Isolation Circuit
IVD	Inductive Voltage Divider
LIA	Lock-in Amplifier
LNA	Low Noise Amplifier
Loc Scr	Local Source
LPF	Low Pass Filter
Lvl Adj	Level Adjuster
Mix	Mixer
MW	Microwave
NMI	National Metrology Institute
NMIJ	National Metrology Institute of Japan
Pos	Position
PS	Phase Shifter
REF	Reference
RF	Radio Frequency
SDOM	Standard Deviation of the Mean
Sig Scr	Signal Source
